# Paraoxonase 1: evolution of the enzyme and of its role in protecting against atherosclerosis

**DOI:** 10.1097/MOL.0000000000000936

**Published:** 2024-06-18

**Authors:** Paul Durrington, Handrean Soran

**Affiliations:** a Faculty of Biology, Medicine and Health, Cardiovascular Research Group, University of Manchester; b NIHR/Wellcome Trust Clinical Research Facility & Department of Diabetes, Metabolism and Endocrinology, Manchester University NHS Foundation Trust, Manchester, UK

**Keywords:** atherosclerosis, HDL, lactones, LDL, paraoxonase 1

## Abstract

**Purpose of review:**

To review the discoveries which led to the concept that serum paraoxonase 1 (PON1) is inversely related to atherosclerotic cardiovascular disease (ASCVD) incidence, how this association came to be regarded as causal and how such a role might have evolved.

**Recent findings:**

Animal models suggest a causal link between PON1 present on HDL and atherosclerosis. Serum PON1 activity predicts ASCVD with a similar reliability to HDL cholesterol, but at the extremes of high and low HDL cholesterol, there is discordance with PON1 being potentially more accurate. The paraoxonase gene family has its origins in the earliest life forms. Its greatest hydrolytic activity is towards lactones and organophosphates, both of which can be generated in the natural environment. It is active towards a wide range of substrates and thus its conservation may have resulted from improved survival of species facing a variety of evolutionary challenges.

**Summary:**

Protection against ASCVD is likely to be the consequence of some promiscuous activity of PON1, but nonetheless has the potential for exploitation to improve risk prediction and prevention of ASCVD.

## INTRODUCTION

Since the discovery of serum paraoxonase 1 as a serum organophosphate esterase in 1953 [1], substantial evidence has accrued linking its activity inversely with the risk of atherosclerotic cardiovascular disease (ASCVD) [2^▪▪^]. The following timeline summarises some of the key events. 

**Box 1:**
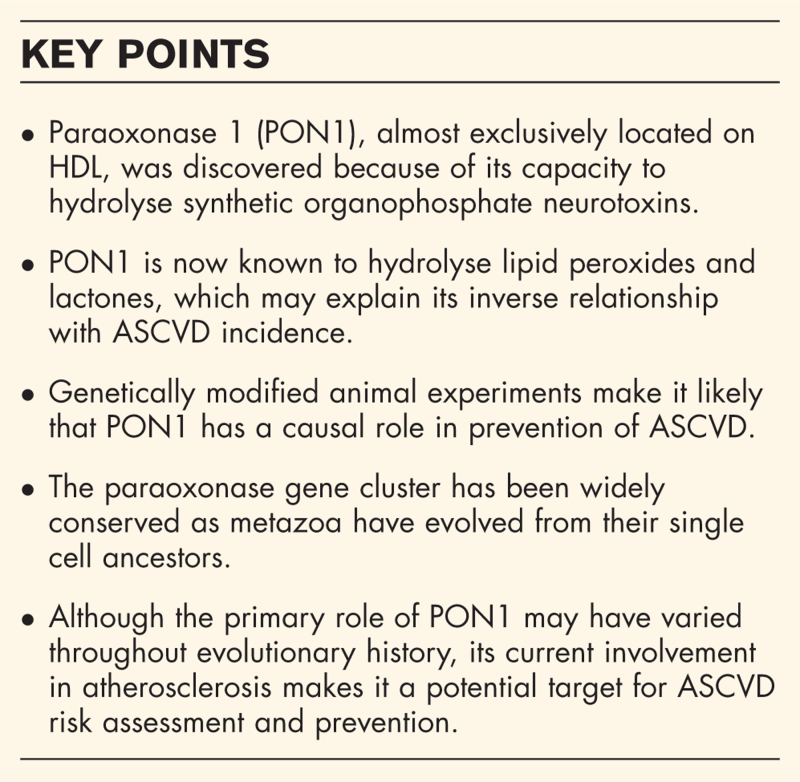
no caption available

## TIMELINE OF SOME OF THE KEY DISCOVERIES LEADING TO THE CONCEPT THAT PON1 PROTECTS AGAINST ATHEROSCLEROTIC CARDIOVASCULAR DISEASE

**1953:** Discovery in human serum of ‘A’esterases hydrolyzing organophosphates and aryl esters [1]. A dispute arose subsequently about whether these activities were due to one or two different enzymes, which was resolved in favour of a single enzyme named as paraoxonase (PON1). There followed extensive studies of its role in toxicology and the discovery on the basis of substrate specificity of its two major polymorphisms, now known to occur as the result of whether glycine(Q) or arginine (R) is present in position 192 of its amino acid sequence [3,4]. This polymorphism is also designated rs662.

**1985:** Discovery that PON1 was located on HDL [5].

**1986:** Serum PON1 activity decreased in myocardial infarction survivors [6].

**1991:** Lower serum PON1 activity in familial hypercholesterolaemia and diabetes mellitus reported [7].

**1991:** First report that PON1 partially purified from HDL prevented the accumulation of lipid peroxides on LDL under oxidizing conditions [8,9].

**1995:** PON1 prevents minimally oxidised LDL-induced migration of human blood monocytes through a layer of cultured endothelial cells and decreases oxidation products of phosphatidyl choline [10]. The 192R isooenzyme of PON1 is associated with ASCVD [11].

**1997:** PON1 present in interstitial fluid [12].

**1998:** HDL from homozygotes for the 192R variant of PON1 protects LDL against oxidation less effectively than that from homozygotes for 192Q [13].

**1998:** PON1 inhibits HDL oxidation and preserves its function [12]. PON1, clusterin and apoA1 present in atheromatous placques co-localize and increase with progression [14].

**1999:** Serum PON1 activity low within 2 h of acute myocardial infarction [15].

**2000--2001:***PON1* promoter polymorphism C-107T is a determinant of serum PON1 activity [16].

**2003–2015:** PON1 activity associated with ASCVD incidence in prospective epidemiological studies [17–24].

**2005:** PON1 not only has a wide range of substrates to which it displays esterase activity, but has even greater lactonase activity and may have its evolutionary origin as a lactonase [25]. Its antiatherosclerotic role could thus be as an antioxidative enzyme, as a lactonase or both.

**2011:** PON1 can redistribute from HDL to cell membranes and influence sensitivity to oxidation [26].

**2012:** HDL rich in PON1 can protect LDL against glycation [27].

## PON1 AND ATHEROSCLEROTIC CARDIOVASCULAR DISEASE CAUSALITY

There are numerous studies in which ASCVD cases were reported to have lower PON1 activity measured with paraoxon or phenyl acetate as substrate. Activity of PON1 has generally been found to be a better discriminator than concentration [2^▪▪^]. Most important amongst these are the prospective studies revealing that the association between lower PON1 activity and ASCVD is not the consequence of the ASCVD event or its treatment or due to lower levels being associated with survival, but that it predated the clinical event [2^▪▪^,^17^–^24^]. However, this type of epidemiology cannot prove causation. In theory, Mendelian randomization studies come closer to revealing causation and the *Q192R* gene variant presents an opportunity for this type of investigation. Since 1995, there have been more than 100 reports, which have with considerable consistency demonstrated an association between the 192R (which is less effective than 192Q in decreasing LDL oxidation) and the likelihood of ASCVD [28,29]. However, 192R does still protect against oxidative modification of LDL albeit less effectively than 192Q. So, higher concentrations can overcome its potentially adverse genotype effect. Thus, the effect is smaller than might be expected from in-vitro experiments in which similar concentrations were compared. Another polymorphism, which can influence PON1 activity and which is located in the promoter region of its gene is T(-108)C (originally designated −107) [16]. Despite some case–control studies showing an association with ASCVD [30], particularly in younger people and diabetes, results have been inconsistent even in investigations where PON1 activity was clearly linked with ASCVD [31]. The conclusion must be that too many other factors are involved in determining PON1 activity and/or that larger prospective studies are required.

Animal experiments provide the strongest evidence that the epidemiologically observed inverse relationship between serum PON1 concentration and ASCVD is causal:

(1) Serum PON1 activity varies greatly throughout the animal kingdom. Birds lack serum paraoxonase activity, whereas humans have substantial amounts and rabbits, for example even more. Whereas human HDL protects LDL against oxidative modification, avian HDL fails to do so [32].
(2) *PON1* knockout mice prone to atherosclerosis both induced by diet and apoE deficiency [33,34].
(3) Overexpression of *PON1* in mouse, rat and rabbit models protects against atherosclerosis [35–39].

Despite the evidence that PON1 is involved in atherosclerosis, this has been questioned most vocally because highly purified or recombinant PON1 has lost some or all of its capacity to prevent LDL oxidation whereas it does retain much of its hydrolytic activity towards organophosphate substrates such as paraoxon [2^▪▪^]. The difficulty with this argument is that to purify PON1 highly whilst still retaining its activity requires that it is maintained in a lipid (hydrophobic) environment, which is hard to achieve. PON1 is itself responsible for much, if not all of the phospholipase A2 (PLA2) activity of HDL [40]. So, demonstrating that an isolate from HDL purified to the point where it has lost PLA2 activity does not have antioxidant activity [41] does not really deny that PON1 is key to the antioxidant activity of intact HDL. Furthermore, recombinant PON1 (rPON1) has undergone mutation(s) to make it water soluble [2^▪▪^]. That rPON1 does not retain antioxidant activity [26] has been challenged [42]. Whilst improvements to its specific activity to certain substrates can be made [43^▪^], it is likely the hydrolysis of, say, long-chain fatty acyl lipid peroxides, which requires the hydrophobic environment of HDL will still impaired unless a means of extracting rPON1 from microbial fermentation products in a lipid-based system [44] or the antioxidant properties of rPON1 can be awakened by combination with lipid derivatives [45^▪^]. The reduced or absent antioxidant activity of highly purified and rPON1 does, of course, give rise to the notion that for its antioxidant activity PON1 requires other HDL components to be present, such as apolipoprotein A1, phospholipase A2 and LCAT [46]. In all probability, the antioxidant role of PON1 is enhanced by other HDL components in maintaining a stable lipid environment or bringing more hydrophobic substrates within the purlieu of its active site [47].

## EVOLUTION AND MECHANISTIC THEORIES

### PON1 as an organophosphatase?

PON1 can hydrolyse a wide range of organic esters including neurotoxic organophosphates deployed in chemical warfare as nerve gases, such as sarin and soman, and in agriculture as pesticides, such as parathion and diazoxin. These agents exert their toxicity because they bind irreversibly to other esterases, such as acetylcholinesterase, essential for neurotransmission. Serum PON1 represents the first line of defence against organophosphate toxicity. The isoenzymes of PON1 resulting from the common Q192R polymorphism differ in their substrate specificities. Thus, although both have similar activity when phenyl acetate is the substrate, the 192Q alloenzyme is more active diazoxin, whereas the 192 R is more active against paraoxon.

Because exposure to synthetic organophosphate substrates is a recent hazard, it was generally considered that PON1 must have evolved for some reason other than as an organophosphatase. However, a vast range of organophosphates occur naturally and the importance of protection against their potential harm should not be discounted as one explanation for the existence of PON1. The habitat of the earliest hominids was on the shores of the great lakes of Africa, where cyanobacteria (blue-green algae) [48], which can produce large quantities of neurotoxic organophosphates, at times, would have threatened human survival. However, modern man has been present for a mere 6 million years, not long enough to explain the conservation of the paraoxonase family of proteins, the ancestral protein for which may have existed hundreds of millions or even billions of years ago. That *PON1* evolved from *PON2*, its intracellular relative [49], has recently been challenged. From an extensive study of many representatives of the animal kingdom, it was concluded that PON1 did not evolve from *PON2*, but that both *PON1* and *PON2* may have evolved from *PON3*[50^▪▪^]. Beyond that, we know that paraoxonases are not homologous to serine esterases, carboxyesterases or arylesterases and thus do not have similar ancestry [49]. The capacity to synthesise cholinesterases and to respond to acetyl choline dates to before metazoa emerged and the evolution of any recognisable nervous system [51]. The potential for organophosphate toxicity must have been present for at least as long. Organophosphates present in the anaerobic conditions around deep sea hydrothermal vents must have been incorporated into the earliest life forms. So, it may not be too fanciful to consider that the ancestral protein giving rise to paraoxonases may have existed many aeons ago and have long had a role in organophosphate metabolism. Other examples of enzymes with organophosphatase activity conserved across the domains of living organisms are diisopropylfluorophosphatase (DFPase) (eukaryocyte squid) [52], organophosphate hydrolase, organophosphate acid anhydrolase and phosphotriesterase (bacteria) [53] and SsoPox an organophosphatase/lactonase from *Sulfolobus solfataricus* (archea) [54]. Of these, the structure of rePON1 resembles that of squid DFPase. Both are six-bladed propellers with each blade consisting of four β-sheets. Moreover, in both structures, two calcium ions can be found in their central tunnel [52].

Polymorphisms of PON1, by broadening the range of organophosphate neurotoxin resistance, will increase the survival of an exposed population without the need to await a new mutation with greater detoxifying properties, albeit at the expense of individuals with the less favourable variant. Darwinian evolution, if by that is meant natural selection of individuals in whom mutations improving fitness for survival are retained, cannot occur sufficiently rapidly to adapt to sudden changes in the environment and extinction may occur before a successful mutation. A preexisting reservoir of members of a species already adapted is essential. In this context, we have reported that agricultural workers involved in sheep-dipping with diazinon (active metabolite diazinoxon) are less likely to experience neuropsychiatric symptoms if they possess the 192Q PON1 allele associated with higher serum PON1 measured as diazoxonase activity [55].

### PON1 as an antioxidant enzyme

With the advent of photosynthetic organisms, an atmosphere rich in oxygen was created and thus the scene was set for the evolution of life with more rapid metabolism (energized by oxidative respiratory chain phosphorylation) than could be sustained by glycolysis and/or the pentose phosphate pathway (PPP). However, simultaneously, the necessity for protection against the toxicity of oxygen also became essential. Paraoxonases and other antioxidative enzymes would have contributed to that [2^▪▪^,^56^]. Although the contribution of PON1 to mechanisms to protect against oxygen-free radical toxicity has been questioned, particularly since the discovery of its lactonase activity, these roles are not mutually incompatible. One may predominate according to the environmental challenges faced by different species. This is discussed in the next sections.

### PON1 as a lactonase

Elias and Tawfik in a fascinating review have strongly argued that paraoxonases may have evolved, not as esterases, but as lactonases with a promiscuous esterase activity [57]. Although not related in other aspects of their structure, their active site has features more in common with lactonases than esterases. Many single-celled organisms signal to each other by producing lactones, such as N-acyl-homoserine lactone (acyl-HSL), usually when their colony size has reached some critical point (quorum sensing), altering expression of genes regulating such processes as bioluminescence, biofilm formation, virulence factor expression and motility. Just as for a hormone to excite rather than inhibit there must be a process to destroy it after receptor binding (ironically, e.g., acetyl choline and acetyl cholinesterase), so a lactonase could have a role in quorum sensing. PON1 also has the capacity to metabolize homocysteine thiolactone and polyunsaturated fatty acid lactones, which could be implicated in atherogenesis [58,59^▪▪^]. However, other hydrolases are active in the hydrolysis of homocysteine thiolactone [60].

Proteins may be more susceptible to glycation by gluconolactone than by glucose [61]. Apolipoprotein B, particularly that in small, dense LDL, undergoes glycation in the circulation where its concentration probably exceeds that of oxidatively modified LDL (ox-LDL), although the glycation and oxidation may occur in tandem [62–64]. Both ox-LDL and glycated LDL are ligands for macrophage scavenger receptor uptake critical for atherogenesis. The major source of gluconolactone is the PPP. The notion that the antecedent of PON1 was a lactonase gives rise to an interesting speculation about its evolutionary conservation beyond microbes, particularly in tissues where the PPP is highly expressed, such as erythrocytes from which it may leak into the circulation. In the PPP, glucose, after its conversion to glucose 6-phosphate, is shunted away from the glycolysis pathway by conversion to 6-phosphogluconolactone and thence to 6-phosphogluconate and then ribose 5-phosphate. The PPP is the source of NADPH, which is essential for scavenging reactive oxygen species. The presence of oxygen at high concentration in erythrocytes is likely to require generation of considerable quantities of NADPH, accounting for the particularly active PPP in red cells. Glycation of haemoglobin may be a consequence of the generation of large amounts of gluconolactone in the PPP. The presence of intraerythrocytic PON2 with its lactonase activity may be important in protecting against loss of function of haemoglobin and other erythrocyte proteins due to glycation. Extracellular PON1 also has the capacity to hydrolyze lactones. It has been reported that HDL from people in the upper half of the distribution of serum PON1 activity prevents LDL glycation more effectively than that from people with lower levels [27]. In-vivo serum PON1 could protect against glycation of LDL when it comes in contact with red cells.

### PON1 in immunity and infection

The view that PON1 may have a more generalized role in the immune system has been proposed by Camps *et al.*[65] based on their finding of an increase in chemokine (C-C motif) ligand 2 (CCL2) production in PON1 deficiency. CCL2 induces migration and infiltration of immune cells into target tissues in a range of inflammatory disorders, which could include the arterial wall. An apparently quite different role for PON1, which might have selective advantage, is its capacity to inactivate gram-negative bacterial endotoxin [66]. This endotoxin is a lipopolysaccharide, which introduces yet another class of substrates which PON1can hydrolyze with important biological consequences. Interference with quorum sensing by metabolism of acyl-HSLhomoserine lactone by PON1 has also been implicated in resistance to infection with the pathogenic bacterium *Pseudomonas aeruginosa*[67].

### Consensus on the role of PON1

Thus, paraoxonases appear to have diverged from other enzymes early in evolution. They display great substrate promiscuity and their primary function (organophosphatase, antioxidant, lactonase, lipopolysaccharidase) may have been different at various times in evolutionary history and in different classes or even orders of living organisms. A recent extensive report revealed there have been multiple independent expansions and contractions of PON throughout metazoa [50^▪▪^]. In contrast to previous findings [4], the results presented in this study suggest that PON1 or PON3 diverged before PON2 [50^▪▪^].

It is inconceivable that PON1 has evolved to combat death from acute myocardial infarction, a syndrome unreported before the twentieth century. Nonetheless, it is very possible that an enzyme which has provided survival success in some other context might by virtue of its promiscuity protect against ASCVD (‘wide substrate specificity’ might be better terminology than ‘promiscuity’ when considering virtue).

### Potential of PON1 in clinical practice

A pharmacological approach to raising PON1 activity is attractive, but traditionally, it is easier to block rather than activate enzymes. Raising HDL by CETP inhibition was ineffective in preventing atherosclerosis except by its LDL-lowering effect [68]. CETP may be necessary for the transfer of oxidized phospholipid and cholesteryl ester to HDL for PON1 to act on them and the HDL particles created are large [69] and not the smaller, desirable particles rich in PON1 capable of facilitating cholesterol efflux. PON1-rich HDL infusion is probably not a practical possibility, particularly as rePON1, which is easily produced may have little antioxidant capacity (see earlier). Evidence suggests that HDL mimetics, some of which could be given orally, can raise PON1 activity in particles resembling physiological HDL [70,71]. It will also be important to be aware of effects on PON1 of the various antisense oligonucleotides for lowering LDL and triglycerides as they emerge. There also exists the theoretical possibility of raising PON1 activity by promotion of its gene or expression of a gain-of-function variant (but without a polar tag so that it is incorporated physiologically into HDL) [72]. An alternative would be to inhibit myeloperoxidase present on HDL, which opposes the antioxidant activity of PON1 [73].

Bariatric surgery is an effective means of raising serum PON1 activity in excessively obese people with metabolic syndrome [74^▪^]. Simultaneously small, dense LDL, ox-LDL and glycated LDL concentrations decline [75–77].

PON1 has the potential to contribute to the clinical assessment of ASCVD risk. However, continuing uncertainties about identification of the substrate critical in its antiatherosclerotic activity have slowed progress in that direction. Is it important, for example, to employ a long-chain fatty acid peroxide or lactone rather than, say, phenyl acetate or paraoxon as the substrate in an assay? However, whilst discovery of the key substrate(s) in the mechanism by which PON1 protects against atherosclerosis is essential for our understanding of its role, this may not be critical to make use of it clinically. Alkaline phosphatase is one of the most frequently requested and informative biochemical tests in clinical practice, but its physiological role remains obscure and the substrate used in its measurement artificial [78]. Currently, measurement of PON1 hydrolytic activity has generally been more closely associated with ASCVD than PON1 protein concentration, because the specific activity of PON1 is variable, for example, in diabetes [79]. PON1 hydrolyzes phenyl acetate at a much higher rate than paraoxon. The PON1 192 polymorphism does not affect the hydrolysis of phenyl acetate, but does that of paraoxon. On the other hand, if the true physiological role of PON1 is its lactonase activity, then potentially there could be advantages to using lactones, such as dihydrocoumarin or homocysteine thiolactone, as assay substrates [80,81]. However, this has yet to be proven. Undoubtedly too, mistakes, such as the use of plasma rather than serum and inclusion of B esterase and nonspecific hydrolysis in some methods for determination of A esterase (PON1) activity, have led to some confusion. A carefully conducted laboratory investigation using a variety of candidate substrates [82,83,84^▪^,^85^] linked to an epidemiological study is required. A method of measuring PON1 using anion exchange membrane (AEM)-based sensor platform technology has recently been published [86^▪^]. A major justification for continuing to study the value of serum PON1 in ASCVD risk assessment is the discordance between HDL cholesterol measurement and PON1 activity [23,87]. Improvements in the analytical error in measurement of PON1 should also mean that it will contribute more to the proportion of risk explained by multivariate analysis.

Measurement of serum PON1 activity also provides an indication of where the HDL present in individuals is in the spectrum of pro- to anti-inflammatory and pro- to antiatherosclerotic capacity [88–90,91^▪▪^]. Cholesterol efflux capacity is another indicator, but its measurement is more difficult and more prone to error [92]. Because decreased PON1 activity is frequently associated with increased SAA in HDL the ratio of SAA concentration to PON1 activity has been proposed as better index of the type of HDL present than either measurement singly [93]. Using similar logic, the ratio pf PON1 activity to HDL myeloperoxidase could also provide an improved index of ASCVD risk [94].

## CONCLUSION

Serum activity of PON1 present on HDL is inversely associated with ASCVD incidence in humans and in animals *PON1* ablation and overexpression lead to increased and decreased atherosclerosis. The initial observations suggesting the possibility that PON1 might protect against ASCVD were made because PON1 isolated from HDL significantly decreased the susceptibility of LDL to oxidative modification. With the discovery of the lactonase activity of PON1 mechanisms by PON1 might protect against the involvement of lactones in atherogenesis also have to be considered. Regardless of how it protects against ASCVD, PON1 provides a potential additional means of clinical risk assessment and is an indicator of the extent to which HDL has retained its antiatherogenic and anti-inflammatory properties. Furthermore, there is therapeutic potential in the activation of PON1 or inhibition of its antagonists, such as myeloperoxidase also present on HDL.

## Acknowledgements

*None.*

### Financial support and sponsorship

*None.*

### Conflicts of interest

*There are no conflicts of interest.*

## REFERENCES AND RECOMMENDED READING

Papers of particular interest, published within the annual period of review, have been highlighted as:

▪ of special interest
▪▪ of outstanding interest
